# Effectiveness of robot-assisted gait training in children with cerebral palsy: a bicenter, pragmatic, randomized, cross-over trial (PeLoGAIT)

**DOI:** 10.1186/s12887-017-0815-y

**Published:** 2017-03-02

**Authors:** C. Ammann-Reiffer, C.H.G. Bastiaenen, A.D. Meyer-Heim, H.J.A. van Hedel

**Affiliations:** 1 Pediatric Rehab Research Group, Rehabilitation Center for Children and Adolescents, University Children’s Hospital Zurich, Mühlebergstrasse 110, CH-8910 Affoltern am Albis, Switzerland; 2 Children’s Research Center, University Children’s Hospital Zurich, Zurich, Switzerland; 3 Functioning and Rehabilitation, CAPHRI, Department of Epidemiology, Maastricht University, Maastricht, The Netherlands

**Keywords:** Child, Adolescent, Cerebral palsy, Robotics, Walking, Therapy, Cross-over design, Randomized controlled trial

## Abstract

**Background:**

Walking ability is a priority for many children with cerebral palsy (CP) and their parents when considering domains of importance regarding treatment interventions. Partial body-weight supported treadmill training has become an established therapeutic treatment approach to address this demand. Further, new robotic rehabilitation technologies have increasingly been implemented in the clinical setting to allow for longer training sessions with increased step repetitions while maintaining a consistent movement pattern. But the current evidence about its clinical effectiveness in pediatric rehabilitation is weak. The aim of this research project is therefore to investigate the effectiveness of robot-assisted gait training on improvements of functional gait parameters in children with cerebral palsy.

**Methods/Design:**

Children aged 6 to 18 years with bilateral spastic cerebral palsy who are able to walk at least 14 m with or without walking aids will be recruited in two pediatric therapy centers in Switzerland. Within a pragmatic cross-over design with randomized treatment sequences, they perform 5 weeks of robot-assisted gait training (three times per week with a maximum of 45 min walking time each) or a 5-week period of standard treatment, which is individually customized to the needs of the child and usually consists of 1–2 sessions of physiotherapy per week and additional hippotherapy, circuit training as well as occupational therapy as necessary. Both interventions take place in an outpatient setting. The percentage score of the dimension E of the Gross Motor Function Measure-88 (GMFM-88) as primary outcome as well as the dimension D of the GMFM-88, 6-minute and 10-meter walking tests as secondary outcomes are assessed before and at the end of each intervention period. Additionally, a 5-week follow-up assessment is scheduled for the children who are assigned to the standard treatment first. Treatment effects, period effects as well as follow-up effects are analyzed with paired analyses and independent test statistics are used to assess carry-over effects.

**Discussion:**

Although robot-assisted gait training has become an established treatment option to address gait impairments, evidence for its effectiveness is vague. This pragmatic trial will provide important information on its effects under clinical outpatient conditions.

**Trial registration:**

ClinicalTrials.gov: NCT00887848. Registered 23 April 2009.

## Background

Mobility in general as well as the ability to walk is a priority for many children with cerebral palsy (CP) and their parents when considering domains of importance regarding treatment interventions [1]. Parents of children with CP value walking, especially ‘correct’ walking, as a key component of their children’s present and future well-being [2]. Children with poorer walking abilities report a reduced physical well-being [3]. Accordingly, acquiring, retaining or improving gait function in these children is often a main goal of the families and the rehabilitation team.

As training intensity, frequency, specificity and level of repetition with variation play a crucial role in promoting sensomotor learning in patients with disorders of the central nervous system, partial body-weight supported treadmill training (PBWSTT) has become an established therapeutic treatment approach [4–6]. Although two or even three therapists may be needed in severely affected patients to support the movement of the legs and stabilize the pelvis and the trunk, PBWSTT takes an important role in gait rehabilitation of adult patients with a diagnosis of stroke, incomplete spinal cord injury (iSCI), Parkinson’s disease (PD) or multiple sclerosis (MS) [7–11]. There is also an increasing body of evidence that PBWSTT improves walking ability, speed and endurance in children, with most evidence being available for children with mild to moderate cerebral palsy [12–15].

**Fig. 1:**
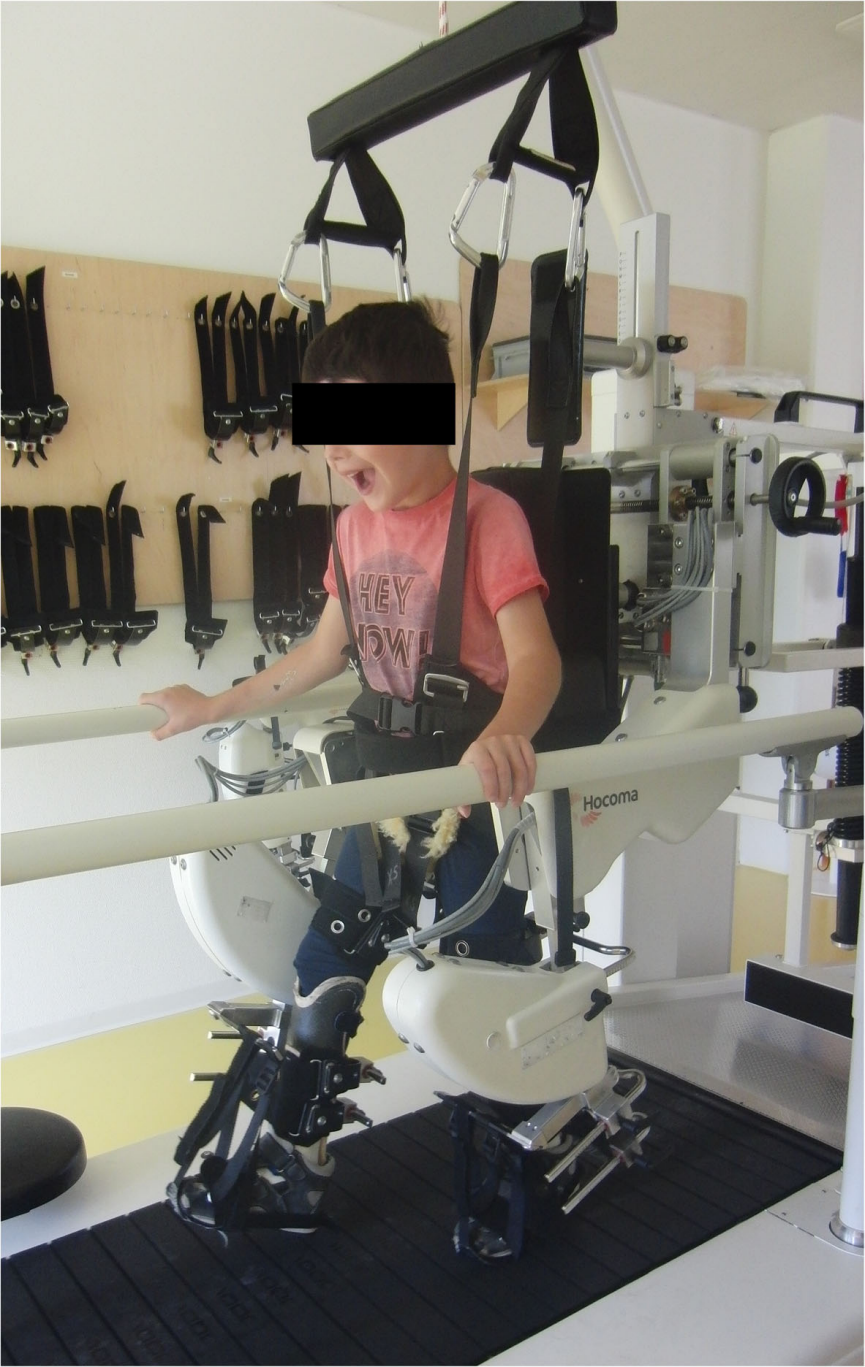
Pediatric robot-assisted gait training with the Lokomat. The Lokomat automates gait therapy on a treadmill by two actuated leg orthoses, which can be individually adapted to the patient’s legs and attached with three cuffs, while the patient is secured by means of a counter system with a harness providing partial body-weight support

To allow for longer training sessions with more repetitions while maintaining a consistent movement pattern and reducing the burden of the therapists, new robotic rehabilitation technologies have emerged during the last 15 years and have increasingly been implemented in the clinical setting [16]. With the addition of virtual reality (VR) scenarios, especially game-based VR, this type of training further offers the patients diversification, fun and challenge [17]. One domain of rehabilitation robots involves driven gait orthoses (DGO) for robot-assisted gait training (RAGT), which have been originally developed for adults and subsequently adapted for children [18–20]. One of these DGOs is the Lokomat (Hocoma AG, Volketswil, Switzerland, Fig. 1), which, by using the pediatric version, allows a training for children starting at an age of approximately 4 years.

RAGT with the Lokomat appears to be effective in improving walking abilities in adult patients with stroke or iSCI [21–23]. A systematic review confirmed these results and showed limited evidence in patients with MS, traumatic brain injury (TBI) or PD [16].

In the last years, RAGT has also been tested and implemented in the pediatric neurorehabilitation setting and has been found to be a feasible and safe therapeutic option [20, 24]. However, the current evidence about the clinical effectiveness of RAGT in pediatric populations is weak. Most published results derive from uncontrolled single case studies or case series [20, 25–28] and the few published controlled trials [29, 30] provide inconclusive results so far.

The aim of this research project is therefore to investigate the effectiveness of RAGT on improvements of functional gait parameters in ambulatory children with cerebral palsy in a pragmatic, randomized cross-over trial.

Our hypothesis is that a 5-week phase of RAGT on the Lokomat in an outpatient setting is superior with regard to the improvement in gait function measured with the dimension E of the Gross Motor Function Measure-88 (GMFM-88) compared to a 5-week phase of standard treatment encompassing conventional physiotherapy lessons in children with spastic CP within a cross-over design with a randomized treatment sequence.

## Methods/Design

The design of the study was developed in accordance with the current (2013) version of the Declaration of Helsinki [31] and the extensions of the CONSORT (Consolidated Standards of Reporting Trials) statement for nonpharmacologic treatment interventions [32] as well as for pragmatic trials [33].

Written informed consent and assent is obtained from all the legal guardians and the child by the PI prior to participation.

### Design and setting

This study is designed as a bicenter, single-blinded, pragmatic, randomized cross-over trial. It is carried out in the outpatient setting of the rehabilitation center of the Children’s University Hospital Zurich in Affoltern am Albis and the Pediatric Therapy Center of the Reha Rheinfelden (Switzerland). Children are randomized to two different pre-specified sequences of interventions. The two interventions are RAGT (T) and usual care (C). A child can be randomized to a T/C sequence (TC-group) or to a C/T/C sequence (CTC-group).

**Fig. 2:**
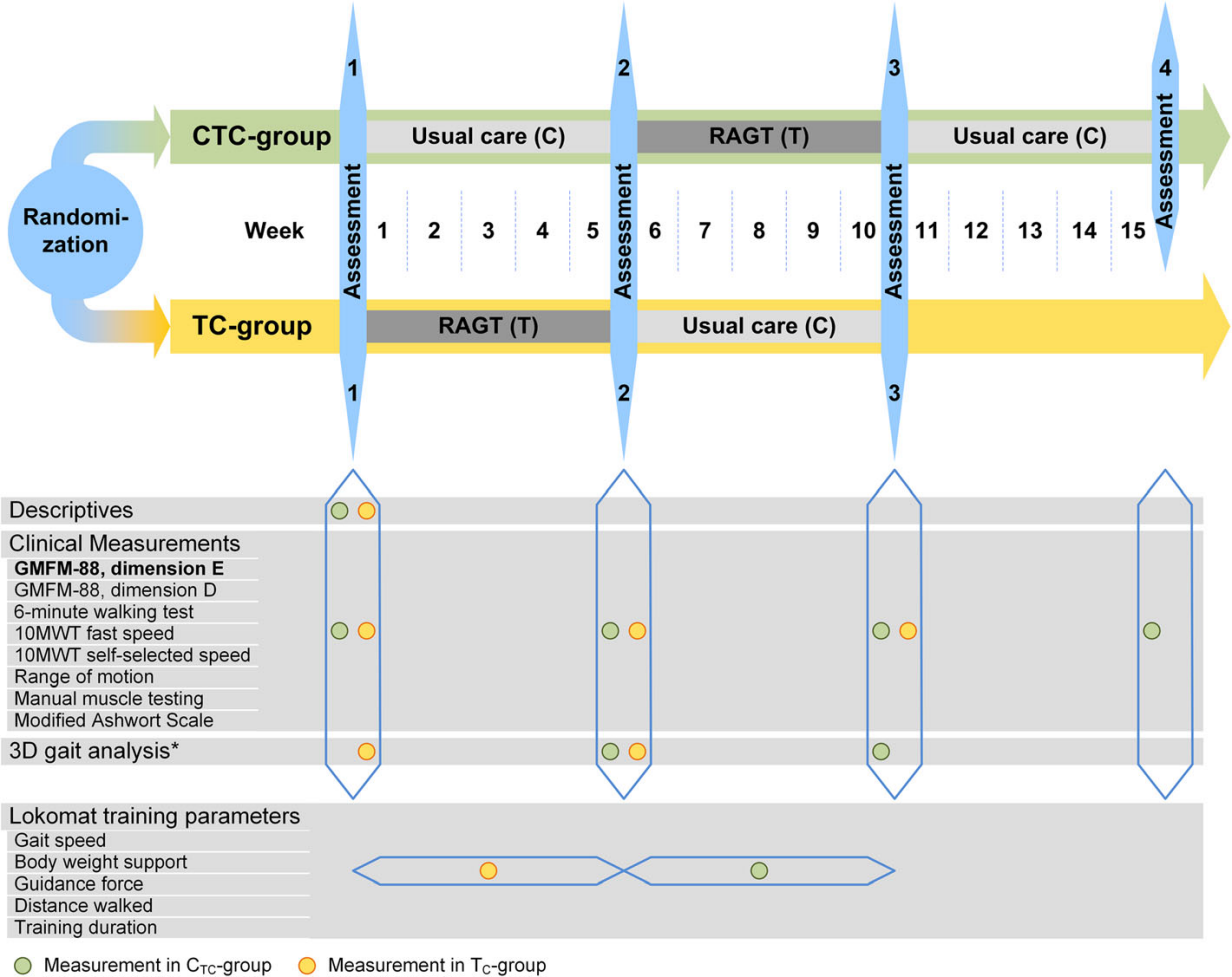
Overview of the outcome measures and the measurement time points per group. Abbreviations: RAGT: Robot-assisted gait training; GMFM-88: Gross Motor Function Measure-88; 10MWT: 10-m walking test; *on an optional basis

The duration of study participation varies dependent on group allocation (Fig. 2): Children allocated to the TC-group start with a first assessment, followed by a 5-week period of RAGT (T-sequence), a subsequent second assessment, a 5-week period of usual care (C-sequence) and a third assessment. Individual study participation will accordingly last 11 weeks for children in the TC-group with three assessment time points.

In contrast to that, children allocated to the CTC-group start with an assessment followed by 5 weeks of usual care (C-sequence) and a subsequent second assessment. This is succeeded by a 5-week period of RAGT (T-sequence), a third assessment, another 5-week period of usual care (C-sequence) and a follow-up assessment. Thus, study participation for children assigned to the CTC-group lasts 16 weeks. The rationale for the differences in study duration and number of assessments between the study groups is that we want to reduce the burden for the children and their families as much as possible while still gaining information on the preservation of a potential effect at follow-up.

### Eligibility criteria

Children aged 6 to 18 years with the diagnosis of a bilateral di- or quadriplegic spastic CP and a Gross Motor Function Classification System (GMFCS) level II-IV, who are able to walk at least 14 meters with or without walking aids, are eligible for study participation. Although we are aware that these criteria encompass a wide range of different motor abilities and developmental states, we consider it important that our study population represents the whole range we encounter in clinical practice. Further, they have to be able to follow instructions and communicate pain or discomfort.

Exclusion criteria are neurosurgery or orthopedic surgery on the lower extremity or trunk within the last 6 months, having participated in another Lokomat training regime within the previous 6 months as well as a change in concomitant treatment within the last 4 weeks before or during the study period. This holds true for all contraindications outlined in the Lokomat manufacturer’s manual, as for example severe contractures (i.e. > 20° knee extension deficit, >40° hip extension deficit), bone fractures, open skin lesions or circulatory problems. Further criteria and their clinical implementation are described in a recently published paper by a Lokomat expert panel [34].

### Recruitment

Patients are recruited by announcement in schools for children with special needs, in pediatric clinics and in outpatient pediatric physical therapy practices. Additionally, we will inform parents of children who have previously trained on the Lokomat and who fulfill the inclusion criteria about the study in a letter. Information about the study is also made available on the website of the two participating centers as well as of the association of Swiss pediatric physiotherapists. Families interested in participating can then take the initiative and contact one of the two rehabilitation centers.

### Randomization, allocation concealment, and blinding

Randomization into the two groups with different intervention sequences is performed using a minimization method with a random factor of 0.9, including the factors severity of impairment (GMFCS-level II or III/IV), age (6–11 years or 11–18 years) and Botulinum Toxin A-treatment (present or absent) in the preceding 6 months. Minimization facilitates balancing even small groups in terms of selected patient factors at all stages of a trial. To ensure that group allocation is concealed for patients and their families as well as everyone who is involved at any stage of the trial, the randomization list is generated by a person not belonging to the study team on www.randomizer.at and stored off site. Directly after inclusion of the patient the principal investigator (PI) seeks information about group allocation through a phone call or an e-mail. As soon as a child is allocated to one of the two groups, the assessments and trainings are planned according to the assigned intervention sequence, taking into account as much as possible the families’ requests and the school schedule of the child.

The rater of the primary outcome and parts of the secondary outcomes is blinded as the performance of the child in these tests is video recorded and scored by means of these videos with the rater being unaware of the date the video has been recorded. The same applies to the statistician.

### Intervention

#### RAGT (T-sequence)

Protocols for PBWSTT as well as RAGT in children are very heterogeneous so far. Session frequencies range from 2 to 5 times per week, session durations vary with most studies reporting 20 to 30 min of treadmill walking per session, and length of treatment varies from 2 weeks to 5 months [34]. As our training protocol prescribes the Lokomat training on an outpatient basis, we consider a session frequency of three times per week with a maximum of 45 min of effective walking time on the Lokomat as feasible and a treatment duration of 5 weeks as clinically adequate.

Trained and experienced therapists, who are used to working with children, perform the Lokomat trainings. The trainings are executed following recently published recommendations [34]. Virtual reality scenarios and other available motivating strategies are used in order to increase the child’s adherence to the training. Body-weight support, gait speed as well as guidance force of the Lokomat are individually adjusted according to the child’s abilities and modified within training sessions and during the course over time. To ensure that uniform standards are applied in both centers, a joint practical training session has been performed and the clinical training standards have been discussed beforehand.

Standard therapies can be continued during the intervention period, but should not include specific walking training and not be changed in frequency. Parents list the type as well as the dosage of concomitant therapy in a personal logbook.

#### Usual care (C-sequence)

Usual care normally comprises 1–2 sessions of physiotherapy per week. Hippotherapy, circuit training as well as occupational therapy can also be elements of standard treatment. These therapies, which are individually customized to the needs of the child, are often implemented in the school setting. The content of the usual care treatment does not specifically address gait training, but rather consists of elements addressing range of motion, tone reduction, balance, activities of daily living, etc. Information about the type and dosage of all therapies is documented in a personal logbook by the parents. As the treatment in our study should represent usual care in clinical practice, we refrained from increasing the dosage for the usual care treatment to a comparable level as for the RAGT intervention.

### Descriptive parameters and measures describing the interventions

An overview of all outcome measures and measurement time points is provided in Fig. 2.

To make sure that the therapists from both centers apply common standards, all performed measures have been thoroughly discussed and trained within and between the teams.

Descriptive and clinical data like diagnosis, walking aids, undergone operations, age, height and weight are recorded in order to describe the characteristics of the included patients. A description and quantification of the RAGT sessions is provided by various measures including the percentage of body weight support, gait speed, guidance force, distance walked and the number and duration of training sessions.

### Primary outcome measure

The primary outcome measure is the percentage score of the dimension E (walking, running, jumping) of the Gross Motor Function Measure-88 (GMFM-88). It assesses the gross motor function with regard to walking ability [35]. The GMFM is a well-validated measurement tool for children with CP, which can be regarded as a gold standard for the assessment of gross motor function in children with CP. The dimension E consists of 24 items, each of which is scored on a 4-point ordinal scale. The children perform the test barefoot, or with shoes and orthoses, if orthoses are used when walking indoors in everyday life. The test is performed without walking aids, regardless of the children’s GMFCS-level. A GMFM-certified therapist instructs the children while the test is simultaneously videotaped. These videos are later scored by a blinded rater, who is also a GMFM-certified therapist. The psychometric properties of the total GMFM-88 are well explored in children with CP. Nevertheless, information on single dimensions is rather sparse. There is limited positive evidence for the responsiveness of dimension E, while evidence on reliability is still unknown because of too little patients included in the reliability studies [36]. A recently published study showed that video rating is a reliable option for the dimensions E as well as D with ICCs of 0.992 and 0.965 for the agreement of life and video scores [37].

### Secondary outcome measures

#### GMFM dimension D

The percentage score of the dimension D (standing) of the GMFM-88 to assess the gross motor function with regard to standing ability. The 13 items are carried out and video rated in the same way as described for the dimension E. As for dimension E, there is evidence for responsiveness of dimension D [35]. Reliability seems to be high, but sample sizes of these studies were rather small to allow definite conclusions [35, 38].

#### Six-minute walking test

The 6-minute walking test (6MinWT) is performed on a 30-meter long corridor with poles at each end. The instruction and encouragement of the child follows a standardized test protocol and the covered distance is noted to one meter [39]. Evidence for the test-retest reliability of the 6MinWT is moderate; its responsiveness has not been established in children with CP so far [36].

#### Ten-meter walking test

Gait speed is assessed with the 10-meter walking test (10MWT), which is performed on a 14-meter long track with the child using the walking aid usually used in daily life. The time needed for the middle 10 meters is measured with a stopwatch, allowing 2 meters for acceleration and deceleration each. The test is performed twice without a break. For the evaluation of fast speed (10MWTfast), the child is instructed to walk as fast as possible but without running, from the first to the last line. The faster out of two trials is used for further analysis. For self-selected speed (10MWTss) the child is instructed to walk in a normal, comfortable speed from the first to the last line and the mean of two trials is calculated. All gait tests are performed with orthoses and walking aids usually used in daily life. Evidence for test-retest reliability of the 10MWTfast is moderate in children with CP [36]. To our knowledge, responsiveness of the 10MWTfast as well as psychometric properties of the 10MWTss has not yet been established in children.

#### Measures of body function

Regarding the child’s body function level, range of motion, modified Ashworth scores, and muscle strength by means of manual muscle testing are evaluated in flexion and extension of the hip, knee and ankle joints [40, 41]. These measures will be used for descriptive purposes only.

One complete assessment block takes maximally 120 min.

Additionally, children perform a 3-dimensional gait analysis (3DGA) on an optional basis before and at the end of the intervention period to capture spatio-temporal and kinematic data of the lower body during walking. The Helen Hayes marker set with 16 markers for the lower body, seven infrared Vicon MX cameras (Oxford Metrix Ltd., Oxford, UK) and two floor-mounted force platforms (AMTI OR 6-7-2000, Advanced Mechanical Technology Inc., Watertown, MA, USA) are used for the 3DGA. The mean of six clean force plate strikes for each foot and each parameter of interest is calculated for further analysis to obtain a reliability level of at least 0.9 for each evaluated discrete gait parameter irrespective of the child’s GMFCS level [42].

### Sample size and power calculation

A sample size calculation for a 2×2 cross-over design was performed. It indicated a sample size of 30 to be sufficient to detect a difference of 3.7%-points in the dimension E-score of the GMFM-88 [43], assuming a standard deviation of 6.8%-points [25], a power of 80% and a significance level of 5%. The 3.7%-points represented the cut-off value that best differentiated between great and not great improvement in motor function in children with CP as judged by their therapists [43]. This number has been increased to 34 to allow for a predicted dropout rate of about 10%.

### Statistical analysis

**Fig. 3:**
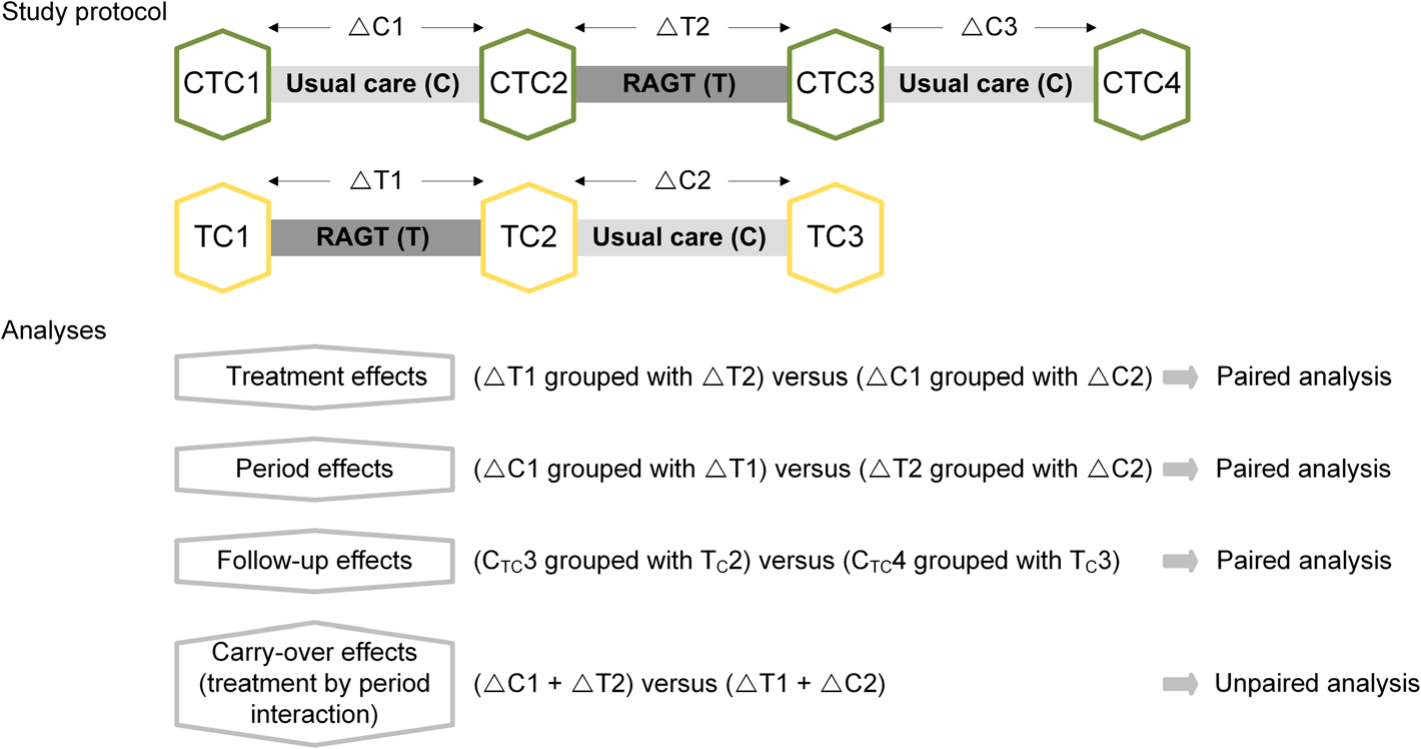
Overview of the study protocol and the statistical analyses. Abbreviations: CTC1: Baseline assessment in CTC-group; TC1: Baseline assessment in TC-group; CTC2: Intermediate assessment in CTC-group; TC2: Intermediate assessment in TC-group; CTC3: End assessment in CTC-group; TC3: End assessment in TC-group; CTC4: Follow-up assessment in CTC-group; ∆C1: Change during usual care in CTC-group; ∆C2: change during usual care in TC-group; ∆C3: Change during follow-up in CTC-group; ∆T1: Change during robot-assisted gait training in TC-group; ∆T2: Change during robot-assisted gait training in CTC-group

All data are electronically filed by the PI using EpiData software with double data entry and range checks for data values (EpiData Association, Odense, Denmark). The statistical analyses are carried out with SPSS24 (IBM Corporation, New York, USA) and datasets of all study participants are analyzed on an intention-to-treat basis. Descriptive parameters of the participants will be presented. Treatment effects, period effects as well as follow-up effects of the two different treatment interventions are analyzed with paired *t*-tests or Wilcoxon signed-rank tests for paired samples, with the treatment effect being our primary end-point. Unpaired two-sample *t*-tests or Wilcoxon rank sum tests for unpaired samples are used to determine whether carry-over effects are present [44]. Ninety-five percent confidence intervals are presented with a two-tailed level of significance set at *p* < 0.05. The applied analyses are schematically listed in Fig. 3.

Subgroup analyses are performed according to impairment level (GMFCS level II vs. GMFCS levels III and IV) and treatment adherence (12 or more training sessions completed vs. less than 12 training sessions), given sufficient numbers within the subgroups.

Missing data will be handled with multiple imputation procedures [45].

## Discussion

RAGT was implemented in the clinical setting of the two participating pediatric neurorehabilitation centers as innovative therapeutic approach almost 10 years ago. It has become an established treatment option in these clinics to address gait impairments in the in- as well as outpatient setting despite the fact that evidence for its effectiveness is vague. As we want to determine the effects of RAGT as it is applied in the clinical setting, we designed this pragmatic trial. The pragmatic trial design allows to evaluate the effects of an intervention under the usual conditions in which it is applied, whereas explanatory trials determine the effects of an intervention under ideal circumstances [46]. Thorpe et al. defined a pragmatic-explanatory continuum, an instrument to determine the extent to which a trial can be viewed as pragmatic or explanatory [46]. Our trial addresses most domains at the rather pragmatic end of this continuum. We make concessions towards a more explanatory approach in two domains: Regarding the flexibility of the experimental design, we standardize frequency, timing and duration of the RAGT intervention and impose some restrictions regarding the co-interventions. A further domain concerns the primary outcome assessment: While we use the dimension E of the GMFM-88, which is a standard outcome in our clinics as well as for the assessment of gross motor functions in children with CP, we deviate from a clear pragmatic approach by videotaping the assessments and including an additional follow-up assessment. We are confident that the chosen trial design allows the evaluation of the effects of RAGT as it is implemented in the daily routine of our clinics, while keeping the additional burden for the participating children and their families as low as possible. This is also the reason why we perform the follow-up assessment only in one group. As participation in this trial still requires a considerable amount of time, we expect some difficulties in recruitment. The study is therefore carried out in two centers, namely the only two centers in Switzerland that offer a pediatric Lokomat training on a regular basis so far. In addition, we designed the study as a cross-over trial. The strength of this design is that each child receives both treatments, but in a random order. Thus, each patient can act as his or her own control requiring only half the number of patients needed in a parallel group design. As variation within a person is usually less than between patients the treatment effect can be estimated with higher precision despite smaller sample sizes [44]. On the other hand, a disadvantage of the cross-over design is the risk of possible carry-over effects, which means that the effect of the treatment in the first period has an effect that lasts into the second period. A carry-over effect leads to an underestimation of the effect of the treatment in the second period. By introducing a washout period between the different treatment periods, this risk could be reduced. Introducing a washout period would lead to an additional assessment. In order to reduce the load of the participants to a minimum we abstained from a washout period being aware that this might influence our results.

Although the 5-week follow-up period is rather short, we opted for this time frame because of the increasing risk of confounding factors that is accompanied by a long time interval due to growth, new therapy approaches, illness, operations, increased school load etc.

### Trial status

August 2009 - November 2018.

## Abbreviations

10MWTfast: 10-meter walking test fast speed
10MWTss: 10-meter walking test at self-selected speed
3DGA: 3-dimensional gait analysis
6MinWT: 6-minute walking test
CP: Cerebral palsy
DGO: Driven gait orthosis
GMFCS: Gross Motor Function Classification System
GMFM: Gross Motor Function Measure
iSCI: Incomplete spinal cord injury
MS: Multiple sclerosis
PBWSTT: Partial body-weight supported treadmill training
PD: Parkinson’s disease
PI: Principal investigator
RAGT: Robot-assisted gait training
TBI: Traumatic brain injury
VR: Virtual reality
